# Misconceptions About Nonverbal Cues to Deception: A Covert Threat to the Justice System?

**DOI:** 10.3389/fpsyg.2020.573460

**Published:** 2020-11-02

**Authors:** Vincent Denault

**Affiliations:** ^1^International Centre for Comparative Criminology, Montreal, QC, Canada; ^2^Department of Communication, Université de Montréal, Montreal, QC, Canada; ^3^Centre for Studies in Nonverbal Communication Sciences, Montreal, QC, Canada

**Keywords:** courtrooms, nonverbal communication, witnesses, deception, credibility

## Introduction

Nonverbal communication is studied by a worldwide community of researchers. Thousands of peer-reviewed papers have been published on the subject (Plusquellec and Denault, 2018). The same holds for deception detection. Unfortunately, misconceptions about nonverbal cues to deception are widespread. The general public holds popular beliefs (The Global Deception Research Team, 2006), unfounded or discredited claims are disseminated on social media and television, and pseudoscientific claims, that is, unfounded or discredited claims presented explicitly or implicitly as having scientific value, are promoted in manuals and seminars (Denault et al., 2015, 2020, Denault et al., submitted).

Misconceptions about nonverbal cues to deception may, at first glance, seem harmless and even entertaining. However, they can have far-reaching consequences. During police investigations, for example, they can result in coercive interrogations and, potentially, false confessions (Leo and Drizin, 2010). During trials, while less discussed within the literature, the consequences can be just as serious, perhaps even more so. Because witness credibility can be largely influenced by demeanor (Denault, 2015), when judges in bench trials (and jurors in jury trials) turn to popular beliefs about deception cues or unfounded, discredited and pseudoscientific claims, the assessment of witness credibility can be distorted. This is significant considering that “credibility is an issue that pervades most trials, and at its broadest may amount to a decision on guilt or innocence” (R. v. Handy, 2002, p. 951) and that decisions of judges are enforceable. When capital punishment is at stake, it can be an issue of life or death (Wilson and Rule, 2015, 2016).

This article aims to highlight the dangers of misconceptions about nonverbal cues to deception during trials. Their popularity among justice and legal practitioners is addressed and, subsequently, their detrimental effect on the assessment of witness credibility. This article ends with recommendations for practitioners, policy makers, and scholars to mitigate the adverse influence of unfounded, discredited and pseudoscientific claims.

## The Popularity of Misconceptions About Nonverbal Cues to Deception

Just as for the general public, several justice and legal practitioners hold popular beliefs about deception cues (e.g., Strömwall and Granhag, 2003; Strömwall et al., 2004; Bogaard et al., 2016). Moreover, despite their considerable authority, several justice and legal practitioners are sympathetic to unfounded, discredited, and pseudoscientific claims. Within law enforcement, this is a well-known problem.

Interviewing techniques promoting nonverbal cues to deception, for example, typically start with a warning to look for clusters of gestures, changes in behaviors, and contradictions between verbal and nonverbal cues (e.g., Walters, 2003; Inbau et al., 2013). The initial warning may, implicitly, convey an impression of scientific rigor or, explicitly, refer to the work of academics. Subsequently, the importance of “body language” is typically touted well-beyond what has been conclusively demonstrated. For example, “any change in a person's constant or normal level of eye contact, which is a timely response and part of a cluster, can be sign of stress and possible deception” (Walters, 2003, p. 134). A final warning sometimes given is that deception cues depend on various factors such as “the perceived seriousness of the offense; the mental and physical condition of the subject; any underlying psychiatric or personality disorders; level of intelligence; degree of maturity; and the extent or absence of social responsibilities” (Inbau et al., 2013, p. 152).

However, research has shown that deception cues are generally faint and unreliable (DePaulo et al., 2003; Sporer and Schwandt, 2007; Luke, 2019; Vrij et al., 2019) and their use has been shown not to significantly improve lie detection accuracy (Hauch et al., 2016; see also Meissner and Kassin, 2004; Bond and DePaulo, 2006; Jordan et al., 2019). Therefore, the initial warning to look for clusters, changes and contradictions becomes trivial, all the more considering accuracy requirements (e.g., that the level of eye contact “can be” a “possible” sign of deception if it is part of a “cluster” of behaviors, a “change” from a “constant” or “normal” behavior, and a “timely” response) negate their practical value. The same holds for the initial and final warnings which, incidentally, also offer an easy response to criticism: you did not obtain the expected results because you did not adequately consider the accuracy requirements and the initial and final warnings.

While unfounded, discredited, and pseudoscientific claims about deception cues promoted to law enforcement in manuals and seminars received attention by both the media (e.g., Hager, 2017; Armstrong and Sheckler, 2019; Smith, 2020) and the academia (e.g., Lilienfeld and Landfield, 2008; Chaplin and Shaw, 2016; Denault et al., 2020), little is known about their promotion to members of the judiciary. An exception comes from Quebec where, for a few years, a number of judges from different courts received talks from proponents of synergology, an approach which, supposedly, makes it possible to “decipher body language.” Proponents of synergology have claimed, among other things, that different gestures have specific meanings, which are not supported by peer-reviewed articles, and promoted concepts similar to the initial and final warnings of interviewing techniques promoting nonverbal cues to deception. Training centers in synergology are located in various countries, including Canada, France, Switzerland, and Spain (for a critical evaluation of synergology, see Denault and Jupe, 2017; Jupe and Denault, 2019; Denault et al., 2020). In other words, unsubstantiated, discredited, and pseudoscientific claims about deception cues can, in the absence of adequate policies, find their way into courtrooms.

## The Detrimental Effects of Misconceptions About Nonverbal Cues to Deception

In countries with adversarial justice systems (e.g., Canada, United States), rules of evidence and procedure foster, to some extent, the use of false beliefs about deception cues and unsubstantiated, discredited, and pseudoscientific claims. During bench trials, for example, judges have to establish the facts to which laws are applied. Essentially, they observe and listen to witnesses and, subsequently, assess their credibility. Based on the witnesses' credibility, judges will give more or less weight to their testimony. This is how judges will often decide what happened when witnesses have different accounts of a same event (Paciocco, 2010; Bell, 2013).

Unfortunately, in several jurisdictions, even if judges are legally authorized to use the witnesses' demeanor to assess their credibility (Mattox v. United States, 1895; Coy v. Iowa, 1988; P. (D.) v. S. (C.), 1993), evidence-based workshops or seminars to mitigate the impact of misconceptions about nonverbal cues to deception are not mandatory. In addition, expert evidence on credibility assessment is generally prohibited. As the Supreme Court of Canada points out, “the issue of credibility is an issue well within the experience of judges and juries and one in which no expert evidence is required” (R. v. Béland, 1987, p. 399). This is in keeping with the Supreme Court of the United States' ruling that jurors “are presumed to be fitted for it by their natural intelligence and their practical knowledge of men and the ways of men” (Aetna Life Ins. Co. v. Ward, 1891, p. 88; United States v. Scheffer, 1998, p. 313). Therefore, it is not uncommon for judges in bench trials (and jurors in jury trials) to turn to popular beliefs about deception cues or unfounded, discredited and pseudoscientific claims.

For example, in a 2019 decision, a judge of the Supreme Court of British Columbia gave little weight to a testimony because, among other things, “As he [the witness] gave his evidence, I observed him to cough, fidget, scratch his neck and at times appear quite nervous” (Garib v. Randhawa, 2019, p. 23). However, research has shown, unequivocally, that those behavioral cues are invalid deception cues (DePaulo et al., 2003; Sporer and Schwandt, 2007; Luke, 2019; Vrij et al., 2019). In a 2020 judgement, on the issue of voluntariness and understanding of a guilty plea, a judge of the Ontario Court of Justice wrote that “assessing body language and making eye contact can be of great assistance in deciding whether or not to accept a guilty plea, as well as weighing and making determinations about sentencing submissions” (R. v. Kerr, 2020, p. 5). However, research has not shown the existence of a body movement or a facial expression to confirm or disconfirm someone is remorseful (Bandes, 2014, 2016). These are just two among many examples (for more examples, see Denault, 2015; Denault and Dunbar, 2019).

However, while written judgments show, in practice, how judges sometimes use misconceptions about nonverbal cues to deception during trials, they are likely the “tip of the iceberg” because the influence of nonverbal communication in face to face interactions occurs much outside of conscious awareness (Goldin-Meadow and Alibali, 2013; Todorov et al., 2015; Hall et al., 2019). And depending on the court's jurisdiction, even if judges “consciously” observe nonverbal cues to deception, they are not required to mention them in their decisions (R. v. Burns, 1994; Cojocaru v. British Columbia Women's Hospital Health Centre, 2013). Therefore, the detrimental effects of both popular beliefs about deception cues and unfounded, discredited and pseudoscientific claims is difficult to measure. In other words, misconceptions about nonverbal cues to deception are a covert threat to the justice system. This is all the more worrisome considering that, even if they are mentioned in written judgments, the assessment of witness credibility is rarely reviewed by appellate courts because, amongst other thing, they cannot “see and hear” the witnesses as judges previously did (Timony, 2000; Denault, 2015). Therefore, judges receive very little feedback and, as a consequence, could read manuals, and attend seminars promoting unfounded, discredited and pseudoscientific claims, all in good faith, throughout their career, without ever being told that, in fact, what they learned is unproven and amounts to nothing more than “junk science” (DeMatteo et al., 2019; Neal et al., 2019).

## Recommendations for Practitioners, Policy Makers, and Scholars

The justice system is a pillar of democracy for societies based on the rule of law. However, for the public to turn to courts when injustice occurs, public trust is fundamental. Unfortunately, misconceptions about nonverbal cues to deception are used for the assessment of witness credibility. Honest witnesses are sometimes believed to be dishonest and dishonest witnesses are sometimes believed to be honest and, as a consequence, parents in family trials can wrongfully lose their children's custody and defendants in criminal trials can wrongfully lose their liberty or their life. This can seriously jeopardize public trust in the justice system. However, a number of measures can be taken in an attempt to mitigate the adverse influence of unfounded, discredited and pseudoscientific claims.

For example, law degrees should incorporate courses on legal psychology and interpersonal communication. Waiting for lawyers to become members of the judiciary to introduce them to these subjects, expecting them to change their years old habits overnight, is irresponsible, if not delusional. Legislative changes should be made to forbid the delivery of courses promoting unfounded, discredited, and pseudoscientific claims to justice and legal practitioners. And justice and legal practitioners should be advised on how to initially assess the scientific quality of manuals and seminars of interest to them. For example, are the instructors “body language experts” or active researchers affiliated with scholarly institutions? Are the claims made during the seminars published in “international bestseller books” or in peer-reviewed publications? Are the seminars promoted using extravagant claims (e.g., “Learn to read people like a book”), appeals to authority (e.g., “We trained FBI and CIA officers”), and anecdotal evidences (e.g., “A terrorist was spotted using our approach”)?

Scholars, on the other hand, should conduct deception research also with members of the judiciary, not only law enforcement, and publish articles in law journals. Changing court culture takes time, but judges regularly turn to law journals for their decisions rather than peer-review articles because unlike the latter, expert testimony is not necessarily required for the former (Hesler, 2002). Furthermore, scholars should actively promote scientific knowledge to justice and legal practitioners, respond to their questions and concerns, and stand up to unfounded, discredited and pseudoscientific claims. While science deniers sometimes turn to ad hominem attacks, and even legal threats (Dance, 2019; Jarry, 2019; Denault et al., 2020), speaking publicly about the importance of science within the justice system is of paramount importance to prevent miscarriages of justice.

## Author Contributions

The author confirms being the sole contributor of this work and has approved it for publication.

## Conflict of Interest

The author declares that the research was conducted in the absence of any commercial or financial relationships that could be construed as a potential conflict of interest. The reviewer JM declared a past co-authorship with the author VD to the handling editor.
